# Cost-effectiveness of a 21-gene recurrence score assay versus Canadian clinical practice in women with early-stage estrogen- or progesterone-receptor-positive, axillary lymph-node negative breast cancer

**DOI:** 10.1186/1471-2407-12-447

**Published:** 2012-10-02

**Authors:** Malek B Hannouf, Bin Xie, Muriel Brackstone, Gregory S Zaric

**Affiliations:** 1 Department of Epidemiology and Biostatistics. Schulich School of Medicine and Dentistry, University of Western Ontario, London, Canada; 2 Department of Obstetrics & Gynaecology, Schulich School of Medicine and Dentistry, University of Western Ontario, London, Canada; 3 Department of Oncology, Schulich School of Medicine and Dentistry, University of Western Ontario, London, Canada; 4 Department of Surgery, Schulich School of Medicine and Dentistry, University of Western Ontario, London, Canada; 5 Richard Ivey School of Business, University of Western Ontario, 1151 Richmond St, London, N6C 1A4, Canada

**Keywords:** Breast cancer, Chemotherapy, Cost-effectiveness, 21-gene recurrence score assay

## Abstract

**Background:**

A 21-gene recurrence score (RS) assay may inform adjuvant systematic treatment decisions in women with early stage breast cancer. We sought to investigate the cost effectiveness of using the RS-assay versus current clinical practice (CCP) in women with early-stage estrogen- or progesterone-receptor-positive, axilliary lymph-node negative breast cancer (ER+/ PR + LN- ESBC) from the perspective of the Canadian public healthcare system.

**Methods:**

We developed a Markov model to project the lifetime clinical and economic consequences of ESBC. We evaluated adjuvant therapy separately in post- and pre-menopausal women with ER+/ PR + LN- ESBC. We assumed that the RS-assay would reclassify pre- and post-menopausal women among risk levels (low, intermediate and high) and guide adjuvant systematic treatment decisions. The model was parameterized using 7 year follow up data from the Manitoba Cancer Registry, cost data from Manitoba administrative databases, and secondary sources. Costs are presented in 2010 CAD. Future costs and benefits were discounted at 5%.

**Results:**

The RS-assay compared to CCP generated cost-savings in pre-menopausal women and had an ICER of $60,000 per QALY gained in post-menopausal women. The cost effectiveness was most sensitive to the proportion of women classified as intermediate risk by the RS-assay who receive adjuvant chemotherapy and the risk of relapse in the RS-assay model.

**Conclusions:**

The RS-assay is likely to be cost effective in the Canadian healthcare system and should be considered for adoption in women with ER+/ PR + LN- ESBC. However, ongoing assessment and validation of the assay in real-world clinical practice is warranted.

## Background

In 2011, an estimated 23,200 women in Canada will be diagnosed with breast cancer [1]. Approximately half of them will be diagnosed with early-stage estrogen- or progesterone-receptor-positive, axillary lymph-node negative breast cancer (ER+/ PR + LN- ESBC) [2]. Standard care for these patients usually includes local therapy (surgery with or without radiation) followed by adjuvant systematic therapy such as endocrine therapy alone (tamoxifen or aromatase inhibitors) or chemotherapy followed by endocrine therapy [3]. Canadian guidelines specify that a patient’s risk of recurrence can be classified as low, intermediate or high and that adjuvant chemotherapy may be added when the benefits of treatment outweigh toxicities of therapy [4]. However, evaluating the risks and benefits of chemotherapy based on the Canadian guidelines is difficult because the histopathologic measures that inform the guidelines are not accurate predictors of risk or benefits of chemotherapy [4-8]. A validated software program Adjuvant!Online (AOL) has been developed that projects outcomes at 10 years to assist oncologists in adjuvant decision-making process. However, AOL is also based on histopathologic measures.

The 21-gene recurrence score assay (Oncotype DX) produces a “tumour signature” reflecting tumour biology and risk of relapse [7,9]. An algorithm produces a continuous variable known as the “recurrence score” (RS) reflecting prognosis, which ranges from 1 (lower risk) to 100 (higher risk), based on the expressions of the 21 genes isolated from tumour samples. Women with a score of less than 18 have a low risk of recurrence and typically have good outcomes from endocrine therapy alone , whereas those with a score of 31 or more have a high risk of recurrence and gain the largest expected benefit from the addition of chemotherapy to endocrine therapy. Women with a score between 18 and 30 have an intermediate risk and do not appear to have a large benefit from chemotherapy but the uncertainty in the estimate cannot exclude a clinically important benefit [9,10].

The prognostic and predictive value of the RS-assay in women with ER+/PR + LN- ESBC was evaluated in retrospective analyses of the National Surgical Adjuvant Breast and Bowel Project (NSABP) chemotherapy-tamoxifen trials (B-14 and B-20) [7,9,11] in the United States. It was shown that among ER+/PR + LN- ESBC patients, approximately, 51% had a low RS, 22% a intermediate RS, and 27% a high RS [7,9,11]. The assay was found to be more accurate than histologic measures alone in predicting the likelihood of breast cancer recurrence (both loco-regional [11] and distant recurrence [7,9]) and patient survival within 10 years of initial diagnosis [9], as well as benefit from adjuvant chemotherapy [9,11]. Additionally, clinical significance of the RS-assay has been reported in the Asian population [12].

In 2007 the RS-assay was recommended in the National Comprehensive Cancer Network and American Society for Clinical Oncology guidelines as “evidence-based” to guide the use of adjuvant chemotherapy in all women with ER+/ PR + LN- ESBC [13,14]. Public coverage of the 21-gene assay is limited and inconsistent across Canada. However, the use of the test with reimbursement mechanisms is likely increasing. It is available in Ontario through “out-of-country health services” which requires a request from an oncologist and pre-approval [15,16]. In 2010 the Ontario Health Technology Advisory Committee (OHTAC) recommended that the assay be made available “within the context of a field evaluation” [17]. It is also available in a limited fashion in British Columbia and Quebec [16]. The test is not widely used and in 2010 less than 1000 patients received the test across Canada [16] but few field evaluations to establish its impact on Canadian practice are ongoing in British Colombia and Ontario.

According to the Annual Report Card of the Cancer Advocacy Coalition of Canada, the RS-assay will cost $4,000 CAD per patient including all Canadian system expenses [15]. Previous cost-effectiveness analyses of the RS-assay in women with ER+/ PR + LN- ESBC in the US [18,19], Japan [20,21], Israel [22] and Canada [23,24] suggested that it is likely to be cost saving or cost effective in this patient group. However, findings from studies in Israel [22] and Japan [19,20] cannot be extrapolated to the Canadian context because of possible variations in clinical practice and different approaches to pricing and reimbursement. Additionally, analyses from the US [18,19] and Canada [23,24] did not use all relevant data and suffer from other limitations as indicated elsewhere [25].

Generation of recommendations for Canadian clinical practice guidelines regarding the use of RS-assay requires a comprehensive health economic evaluation of the assay in the Canadian setting. The purpose of this study was to conduct a cost-effectiveness analysis of the RS-assay versus current clinical practice (CCP) regarding adjuvant chemotherapy treatment in women with ER+/ PR + LN- ESBC from the perspective of the Canadian healthcare system.

## Methods

### Overview of model-structure

**Figure 1:**
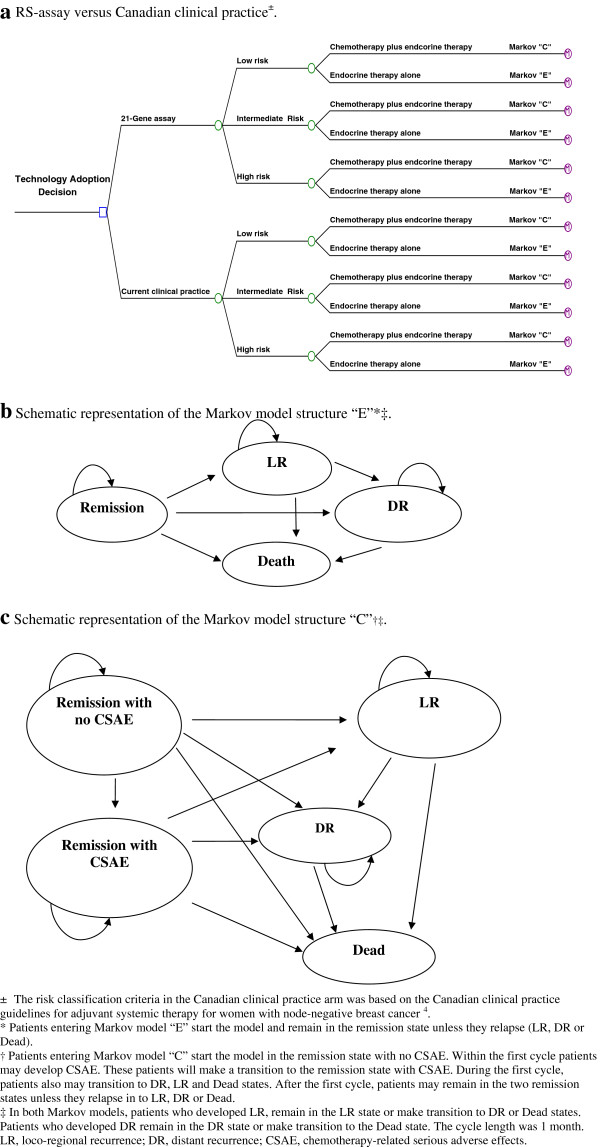
**Decision model for early stage breast cancer.a** RS-assay versus Canadian clinical practice^±^. **b** Schematic representation of the Markov model structure “E”*‡. **c** Schematic representation of the Markov model structure “C”†‡.± The risk classification criteria in the Canadian clinical practice arm was based on the Canadian clinical practice guidelines for adjuvant systemic therapy for women with node-negative breast cancer [4] * Patients entering Markov model “E” start the model and remain in the remission state unless they relapse (LR, DR or Dead). † Patients entering Markov model “C” start the model in the remission state with no CSAE. Within the first cycle patients may develop CSAE. These patients will make a transition to the remission state with CSAE. During the first cycle, patients also may transition to DR, LR and Dead states. After the first cycle, patients may remain in the two remission states unless they relapse in to LR DR or Dead. ‡ In both Markov models, patients who developed LR, remain in the LR state or make transition to DR or Dead states. Patients who developed DR remain in the DR state or make transition to the Dead state. The cycle length was 1 month. LR, loco-regional recurrence; DR, distant recurrence; CSAE, chemotherapy-related serious adverse effects.

We developed a decision analytic model (Figure 1) to project the lifetime clinical and economic consequences of ER+/ PR + LN- ESBC under two different treatment strategies. The model begins with a decision to use the RS-assay or to continue with CCP (Figure 1a). We assumed that each strategy (RS or CCP) classifies patients into three risk levels (low, intermediate and high) and corresponding treatment regimens (endocrine therapy plus chemotherapy or endocrine therapy alone). Patients receiving endocrine therapy alone entered model “E” (Figure 1b) and those receiving chemotherapy plus endocrine therapy entered model “C” (Figure 1c).

Model “E” simulated monthly transitions among the following four distinct health states: (1) remission; (2) loco-regional recurrence (LR); (3) distant recurrence (DR); (4) death. Model “C” simulated monthly transitions among the following five distinct health states: (1) remission with no chemotherapy-related serious adverse effects (CSAE); (2) remission with CSAE; (3) LR; (4) DR; (5) death.

We used a lifetime horizon and half cycle correction [26]. Future costs and benefits were discounted at 5% annually following Canadian guidelines [27]. Data collection and analysis involving Manitoba administrative databases (including the Manitoba Cancer Registry, the Hospital Discharge Database, the Physician Claims Database and the Drug Program Information Network) were approved by the University of Manitoba Health Research Ethics Board.

### Risk distribution and transition probabilities

The Manitoba Cancer Registry is a provincial database that contains records for more than 99.5% of all cancer patients in Manitoba [28]. Information on breast cancer staging, based on the American Joint Commission on Cancer (version 5), has been collected for breast cancers diagnosed since January 1995 [29]. We used the Registry to identify a study cohort consisting of all pre-menopausal (defined as age <50 years) and post-menopausal (age ≥50 years) women living in Manitoba diagnosed with ER+/ PR + LN- ESBC (stage I/II) during the period from January 1, 2000 to December 31, 2002. Although data on human epidermal growth factor receptor 2 (HER2) status were not collected by the registry during this time frame, the majority of these women are likely HER2 negative since women with HER2 positive are only found in approximately 10% to 15% of endocrine positive breast cancers such as those in our study population [30-34]. We used data from women diagnosed during this period so that a long follow up period would be available. Seven-year follow-up information from the time of diagnosis was available for each patient. This included breast cancer recurrence (LR and DR) and treatments (surgery, radiation therapy, endocrine therapy and chemotherapy). We linked the study cohort identified using the Registry with administrative data held by Manitoba Health and Healthy Living including the Hospital Discharge Database, the Physician Claims Database and the Drug Program Information Network. To protect confidentiality, the linkage in this study was performed, via scrambled health number, using anonymized versions of these databases.

To verify that the proportion of women who received adjuvant chemotherapy in our study cohort would reflect more recent clinical practice regarding adjuvant chemotherapy administration, we examined a second cohort, consisting of all women diagnosed between January 1, 2003 and December 31, 2005 (Table 1). We did not find the proportion receiving adjuvant chemotherapy to differ between the two time periods (chi-square test, level of significance of 0.05) and thus used the earlier time period with longer follow-up data to parameterize the model.

**Table 1:** Proportion of patient population receiving adjuvant chemotherapy by diagnosis time period and menopausal status

**Diagnosis time period**	**No. of women who diagnosed with ER + or PR + LN- ESBC**		**No. of women received adjuvant chemotherapy (%)**			
	**Pre-menopausal women**	**Post-menopausal women**	**Pre-menopausal women**	**2000-2002 vs. 2003-2005**	**Post-menopausal women**	**2000-2002 vs. 2003-2005**
				**ρ value†**		**ρ value†**
2000 − 2002	109	389	74 (69)	.88	73 (18.8)	.7
2003 − 2005	106	506	71 (67)		90 (17.7)	

† Chi-square test.

For the CCP model we estimated the risk distribution and proportion receiving chemotherapy within each risk level (Table 2). According to the Canadian clinical practice guidelines, risk can be specified on the basis of tumour size, histologic or nuclear grade, and lymphatic and vascular invasion [4]. The Manitoba Cancer Registry collects this information with the exception of lymphatic and vascular invasion. Given the significant correlation between tumour size and lymphatic and vascular invasion [35], we classified pre- and post-menopausal women for this analysis as belonging to three risk levels (low, intermediate and high risk) on the basis of tumour size and histologic or nuclear grade only. We defined current clinical practice according to the observed administration of adjuvant therapy in the ER+/ PR + LN- ESBC cohort during the study period. We conducted survival analyses using Kaplan-Meier estimates for pre- and post-menopausal women separately, stratified by use of adjuvant chemotherapy, using 7 years of follow up data from the Manitoba Cancer Registry, and used this information to estimate all transition probabilities in the CCP Markov models.

**Table 2:** Parameter estimates and sources

**Variables**	**Pre-menopausal Women**		**Post-menopausal Women**		**Duration**	**Distribution used in PSA†**	**Source**
	**Base case value**	**Range tested in sensitivity analyses**	**Base case value**	**Range tested in sensitivity analyses**			
**Risk classification by CCP (%)**							
**High risk**	21.1	15.8 **–** 32.6	22.3	18 **–** 27		Dirichlet	MCR
**Chemotherapy-treated women**	100	85.1 **–** 100	53.8	43 – 64.4		Beta	MCR and PC
**Intermediate risk**	72.6	62.9 **–** 80.6	52.3	47 **–** 57.5		Dirichlet	MCR
**Chemotherapy-treated women**	65.2	53.4 **–** 75.4	14.2	9.9 – 20		Beta	MCR and PC
**Low risk**	6.3	0 – 10	25.4	21.2 **–** 30.2		Dirichlet	MCR
Chemotherapy-treated women	16.7	10 – 20	3.4	0 – 10		Beta	MCR and PC
Overall chemotherapy-treated women by CCP (%)	69	60 **–** 83	19	13 – 27.7			MCR and PC
**Risk classification by RS-assay (%)**							
**High risk**	27.7	22.9– 33.1	23.1	18.7 – 28.3		Dirichlet	[9]
**Chemotherapy-treated women**	100	90 – 100	100	90 – 100		Beta	[9]
**Intermediate risk**	19.5	15.4 – 24.4	21.5	17.1 – 26.5		Dirichlet	[9]
**Chemotherapy-treated women**	50	0 – 100	50	0 – 100		Beta	[22,39,40,65]
**Low risk**	52.6	46.9 – 58.3	55.4	49.7 – 61		Dirichlet	[9]
Chemotherapy-treated women	0	0 – 10	0	0 – 10		Beta	[9]
Overall chemotherapy-treated women by RS-assay (%)	37.5	30 – 47.8	33.8	27 – 44.3			[9,22,39,40,65]
**Chemotherapy-related serious adverse effects (%)**	2.5	0 – 10.6	4	0 – 12.3		Beta	MCR and HA
**Health-State Utilities‡**							
Remission state							
Remission on chemotherapy regimen with							
Minor or no toxicity	0.85	−20%	0.783	−20%	6 months	Beta	[51,52,55]
Remission on chemotherapy regimen with							
Major toxicity	0.623	−20%	0.577	−20%	6 months	Beta	[51,52,55]
Remission after chemotherapy regimen	0.872	−20%	0.808	−20%	Life	Beta	[51,54]
Remission on hormonal therapy	0.881	−10% **– +**10%	0.816	−10% **– +**10%	60 months	Beta	[51,52,55]
Remission after hormonal therapy	0.89	−10% **– +**10%	0.824	−10% **– +**10%	Life	Beta	[51,52,55]
Loco-regional recurrence, under treatment	0.623	−10% **– +**10%	0.577	−10% **– +**10%	12 month	Beta	[41,51,52,55]
Loco-regional recurrence, after treatment	0.757	−10% **– +**10%	0.700	−10% **– +**10%	Life time	Beta	[41,51,52,55]
Distant recurrence	0.445	−10% **– +**10%	0.412	−10% **– +**10%	Life time	Beta	[41,51,52,55]
Death state	0		0				
**Cost associated with remission (per month), $**							
First year after diagnosis with ESBC							
Cost of surgery^a^	3390	3000 – 3780	3642	3384 – 3900	One time	LogNormal	PC, HA and CL
Cost of radiation therapy^b^	3410	2737 – 4252	3027	2430 – 3776	One time	LogNormal	PC and CL
Cost of endocrine therapy^c^							
Tamoxifen	12.4	11.6 – 13.2	12.4	11.6 – 13.2	12 months	LogNormal	DPIN
Aromatase inhibitors			156	120 **–** 193	12 months	LogNormal	DPIN
Aromatase + tamoxifen			72	62 **–** 81	12 months	LogNormal	DPIN
Cost of chemotherapy^d^							
Nursing, overhead and administration costs	317.6		317.6		During chemotherapy	LogNormal	CL
Related physician costs	23.4	21.5 – 25.2	23.4	21.5 – 25.2	During chemotherapy	LogNormal	PC
Chemotherapy regimen options							
CMF	478		823		5 months	LogNormal	MCR
AC	806		1918		3 months	LogNormal	MCR
FAC	924		1270		5 months	LogNormal	MCR
TAC	2455		2800		5 months	LogNormal	MCR
Weighted average cost of chemotherapy regimens^e^					5 months	LogNormal	MCR
First three months on chemotherapy	1142		1099		3 months	LogNormal	MCR
Next	419		432		2 months	LogNormal	MCR
Cost of CSAE^f^	1263	978 – 1581	1,750	1376-2168	During chemotherapy	LogNormal	PC, HA and CL
Surveillance^g^							
Low risk	79	47 **–** 111	74	62 **–** 85	12 months	LogNormal	PC
Intermediate risk	93	76 **–** 108	66	60 **–** 68	12 months	LogNormal	PC
High risk	106	78 **–** 133	77	69 **–** 82	12 months	LogNormal	PC
After first year of diagnosis with ESBC							
Cost of endocrine therapy^c^							
Tamoxifen	12.4	11.6 – 13.2	12.4	11.6 – 13.2	48 months	LogNormal	DPIN
Aromatase inhibitors			156	120 **–** 193	48 months	LogNormal	DPIN
Aromatase + tamoxifen			72	62 **–** 81	48 months	LogNormal	DPIN
Surveillance^g^							
Low risk	39	18 **–** 59	33	30 **–** 54	Life time	LogNormal	PC
Intermediate risk	35	32**–** 40	45	38 **–** 53	Life time	LogNormal	PC
High risk	102	65 **–** 126	39	32 **–** 45	Life time	LogNormal	PC
**Cost associated with LR (per month), $**							
First year after LR							
Cost of surgery^a^	3522	889 – 7280	2806	1068 – 3111	One time	LogNormal	PC, HA and CL
Cost of radiation therapy^b^	1098	878 – 1371	2120	1695 – 2651	One time	LogNormal	PC, HA and CL
Cost of endocrine therapy^c^							
Tamoxifen	12.4	11.6 – 13.2	12.4	11.6 – 13.2	12 months	LogNormal	DPIN
Aromatase inhibitors			156	120 **–** 193	12 months	LogNormal	DPIN
Sequential aromatase → tamoxifen			72	62 **–** 81	12 months	LogNormal	DPIN
Cost chemotherapy^d^	278	181 – 619	311	200 – 688	5 months	LogNormal	PC and CL
Surveillance during first year^g^	118	48 – 189	123	64 – 179	12 months	LogNormal	PC
After first year of LR							
Cost of endocrine therapy^c^							
Tamoxifen	12.4	11.6 – 13.2	12.4	11.6 – 13.2	48 months	LogNormal	DPIN
Aromatase inhibitors			156	120 **–** 193	48 months	LogNormal	DPIN
Sequential aromatase → tamoxifen			72	62 **–** 81	48 months	LogNormal	DPIN
Surveillance after first year of LR^g^	98	33 – 162	78	18 – 139	Life time	LogNormal	PC
**Cost associated with DR (per month), $**							
First year after DR							
Hospitalization cost	841	138 – 253	1569	185**–** 3177	12 months	LogNormal	HA and CL
Physicians cost	247	64 – 431	353	205 – 501	12 months	LogNormal	PC
Drugs cost	19	5 – 34	83	29 – 134	12 months	LogNormal	DPIN
After first year of DR							
Hospitalization cost	1293	146 – 3014	783	72 – 1618	Life time	LogNormal	HA and CL
Physicians cost	204	86 – 322	183	62 – 337	Life time	LogNormal	PC
Drugs cost	52	5 – 121	100	33 – 167	Life time	LogNormal	DPIN

**†** Beta distribution was used for other probability parameter estimates not included in this table.

**‡** The baseline utility for post-menopausal women aged 50 to 80 was 0.824 and for premenopausal women aged 20 to 49 was 0.89 [51]. We derived utilities for each state by multiplying these baseline utility values by utility estimates for women with breast cancer [41,52-54], consistent with methodology as described by Fryback [55].

^a^ Cost of breast cancer surgery: We used the Hospital Discharge Database and the Physician Claims Database to estimate the mean cost of hospitalization due to any breast cancer surgery (including one day hospitalization and using the ICD-9-CM procedure codes for a hospital abstract) within one year after diagnosis with ESBC and LR by menopausal status.

^b^ Cost of radiation therapy: Cost of radiation therapy included cost of radiation therapy–related physician claims in addition to administrative cost. We used the Physician Claims Database to estimate the mean cost of radiation therapy–related physician claims (using the tarrif code for a medical claim) within one year after diagnosis with ESBC and LR by menopausal status. Administrative costs were derived from the cost list for Manitoba health services.

^c^ Cost of endocrine therapy: We used the Drug Program Information Network to estimate the mean cost of tamoxifen and aromatase inhibitors by menopausal status (using the drug identification number for a drug claim) within the time periods, between diagnosis with ESBC and before any relapse, and diagnosis with LR and before any relapse.

^d^ Cost of chemotherapy: Nursing, overhead and administration costs were derived from the cost list for Manitoba health Services. We used the Physician Claims Database to estimate the mean cost of chemotherapy–related physician claims costs (using the tarrif code for a medical claim) within one year after diagnosis with ESBC and LR by menopausal status. Chemotherapy regimens costs were estimated based on the market prices as of May 2010.

^e^ Weighted average cost of adjuvant chemotherapy regimens: We calculated the average cost of adjuvant chemotherapy regimens weighted to the observed proportion use of anthracyclines and taxanes by menopausal status. Weighted average cost of adjuvant chemotherapy regimens = proportion of women received non-anthracyclines containing adjuvant chemotherapy × cost of CMF + proportion of women received anthracyclines containing adjuvant chemotherapy (no added taxanes) × cost of AC + proportion of women received anthracyclines and taxanes containing adjuvant chemotherapy × cost of TAC.

^f^ Cost of CSAE: We used the Hospital Discharge Database and the Physician Claims Database to estimate the mean cost associated with hospitalizations due to any of the eight diagnoses which were considered CSAE among women who develop CSAE. We stratified the analysis by menopausal status.

^g^ Cost of surveillance: We defined the cost of breast cancer surveillance as the incremental cost of health care utilization (medical claims) after diagnosis with ESBC versus the time before diagnosis. We used the Physician Claims Database to collect medical claims for both post- and pre-menopausal women, within 3 years before and 7 years after diagnosis with ESBC. We estimated the mean cost of medical claims by menopausal status within 3 years before diagnosis in order to reflect the usual cost of health care utilization. We calculated the incremental mean cost of health care utilization by menopausal status during the period from diagnosis with ESBC and before any relapse (excluding cost of claims related to surgery, radiation therapy, chemotherapy and CSAE) stratified by the time following diagnoses (first year versus later). Similarly, we calculated the incremental mean cost of health care utilization by menopausal status after LR

PSA = probabilistic sensitivity analysis; MCR = Manitoba Cancer Registry: PC = physician claims; HA = hospital abstracts; CL = cost list for Manitoba health services; DPIN = Drug Program Information Network records; ESBC = early stage breast cancer; LR, loco-regional recurrence; DR = distant recurrence; CMF = 6 cycles of cyclophosphamide, methotrexate, 5-fluorouracil; AC = 4 cycles of adriamycin, cyclophosphamide; FAC = 6 cycles of fluorouracil, doxorubicin, cyclophosphamide; TAC = 6 cycles of docetaxel, doxorubicin, cyclophosphamide; CCP = current clinical practice.

For the RS-assay model, we derived the risk distribution and monthly transition probabilities from remission to LR, DR and Death over 10 years within each risk level from retrospective analyses of the NSABP chemotherapy-tamoxifen trials (B-14 and B-20) (Table 2) [9,11]. Investigators from the B-14 and B-20 studies provided Kaplan Meier curves for LR, DR and death events stratified by risk level. To account for menopausal status, we adjusted all transition probabilities derived from these summary statistics based on corresponding risk ratios (for LR, DR and death) comparing pre- to post-menopausal women derived from our studied ESBC cohort. The risk ratios were weighted using the menopausal status balance reported in the B-14 and B-20 trials [9,11].

There is still uncertainty as to whether chemotherapy is necessary for women with intermediate risk. Reported usage in this group varies, including estimates of 56% [36], 50% [37], 47% [38], 38% [39], 33% [22], and 26% [40]. In the base case we assumed that 50% of women in the intermediate risk group would receive adjuvant chemotherapy.

There is no data suggesting that outcomes after first relapse are affected by the primary adjuvant therapy received [41]. Thus, we assumed that transition probabilities following first relapse in the RS-assay model would be the same as those in the CCP model.

To extrapolate beyond the follow-up period of the ESBC cohort and the clinical trials used for this study, we assumed that the observed average monthly transition probabilities from remission to LR, DR and Death during the last observed year of follow-up would be constant over the extrapolated lifetime. We used female age-adjusted life tables for Manitoba to adjust the probabilities from remission to death in order to account for the incremental mortality risk over the extrapolated time [42].

### Adjuvant chemotherapy regimens

In Canada, from 2000–2002, two adjuvant chemotherapy regimens were recommended for women with ER+/ PR + LN- ESBC: (1) 6 cycles of cyclophosphamide, methotrexate, 5-fluorouracil (CMF) or (2) anthracycline-containing chemotherapy regimen such as 4 cycles of doxorubicin (adriamycin), cyclophosphamide (AC) or 6 cycles of fluorouracil, doxorubicin, cyclophosphamide (FAC) [4]. Four cycles of AC has been used preferentially as a component of chemotherapy regimens for the adjuvant treatment of ESBC [43]. Recently, chemotherapy regimens containing taxanes, such as 6 cycles of docetaxel, doxorubicin, cyclophosphamide (TAC), have been recommended for the LN- ESBC population [44].

The majority of adjuvant chemotherapy-treated women in our study cohort received anthracycline-containing adjuvant chemotherapy regimens (Table 3). Information on specific chemotherapy agents (e.g. CMF, AC, FAC, and TAC) was not available. We assumed patients who received non-anthracycline-containing adjuvant chemotherapy regimens received 6 cycles of CMF; that patients who received anthracycline-containing adjuvant chemotherapy regimens with no added taxanes received four cycles of AC; and that patients who received anthracycline and taxane-containing adjuvant chemotherapy regimens received 6 cycles of TAC. Thus, in the base case analysis, we used the weighted average cost of CMF, AC and TAC.

**Table 3:** Characteristics of 489 patients diagnosed during the time period of 2000 to 2002 with ER + or PR + 1–3 LN + ESBC stratified by menopausal status and risk of recurrence using Canadian clinical practice guidelines

**Characteristic**		**Pre-menopausal women (n = 109)**				**Post-menopausal women (n = 389)**				**ρ value†**
		Low risk*	Intermediate risk*	High risk*	**Overall**	Low risk*	Intermediate risk*	High risk*	**Overall**	
		**(n =** **11)**	**(n =** **78)**	**(n =** **20)**	**(n = 109)**	**(n =** **115)**	**(n =** **196)**	**(n =** **78)**	**(n = 389)**	
Age ( years)										
Mean (range)		41.8	43.6	42.7	43	63.4	64	61.8	63	
		(30 – 49)	(29 – 49)	(33–49)	(29–49)	(50–85)	(50 – 88)	(50–86)	(50–88)	
<40		3 (27.3)	17 (21.8)	4 (20)	24 (22)	―	―	―	―	
40 – 49		8 (72.7)	61 (78.2)	16 (80)	85 (78)	―	―	―	―	
50 – 64		―	―	―	―	64 (55.7)	111 (56.6)	53 (68)	228 (58.6)	
≥65		―	―	―	―	51 (44.3)	85 (43.4)	25 (32)	161 (41.4)	
Primary tumour size – no. of women (%)										
<2 cm		11 (100)	51 (65.4)	7 (35)	69 (63.3)	115 (100)	117 (59.7)	17 (21.8)	260 (66.8)	.78
2-5 cm		0	27 (34.6)	11 (55)	38 (34.9)	0	79 (40.3)	55 (70.5)	123 (31.7)	
>5 cm		0	0	2 (10)	2 (1.8)	0	0	6 (7.7)	6 (1.5)	
Receptor status – no. of women (%)										
ER + and PR-		0	11 (14.1)	7 (35)	18 (16.6)	25 (21.7)	54 (27.5)	30 (38.5)	109 (28)	.016
ER- and PR+		0	4 (5.2)	3 (15)	7 (6.4)	1 (0.9)	4 (2.1)	6 (7.7)	11 (2.8)	
ER + and PR+		11 (100)	63 (80.7)	10 (50)	84 (77)	89 (77.4)	138 (70.4)	42 (53.8)	269 (69.2)	
Tumour grade – no. of women (%)										
1		6 (54.5)	14 (18)	1 (5)	21 (19.3)	89 (77.4)	17 (8.7)	1(1.3)	107 (27.5)	.37
2		0	50 (64.1)	5 (25)	55 (50.5)	0	160 (81.6)	21 (26.9)	181 (46.5)	
3		0	5 (6.4)	14 (70)	19 (17.4)	0	6 (3)	53 (68)	59 (15.2)	
Unknown		5 (45.5)	9 (11.5)	0	14 (12.8)	26 (22.6)	13 (6.7)	3 (3.8)	42 (10.8)	
Stage										
I		11 (100)	55 (70.5)	7 (35)	73 (67)	115 (100)	145 (74)	21 (26.9)	281 (72.2)	.56
IIA		0	23 (29.5)	11 (55)	34 (31.2)	0	51 (26)	51 (65.4)	102 (26.2)	
IIB		0	0	2 (10)	2 (1.8)	0	0	6 (7.7)	6 (1.6)	
With Breast-surgery‡ − no. of women (%)		11 (100)	78 (100)	20 (100)	109 (100)	115 (100)	196 (100)	78 (100)	389 (100)	
Breast-conserving surgery		8 (72.7)	51 (65.4)	9 (45)	68 (62.4)	65 (56.5)	113 (57.7)	29 (37.2)	207 (53.4)	.08
Mastectomy		3 (27.3)	27 (34.6)	11 (55)	41 (37.6)	50 (43.5)	83 (42.3)	49 (62.8)	182 (46.6)	
With Radiotherapy‡ − no. of women (%)		7 (63.6)	51 (65.4)	11 (55)	69 (63.3)	62 (54)	109 (55.6)	30 (38.5)	201 (51.7)	.03
With Endocrine therapy‡ − no. of women (%)		5 (45.4)	65 (83.3)	18 (90)	88 (81)	91 (79.1)	165 (84.1)	53 (67.9)	309 (79.4)	.76
Tamoxifen		5 (100)	49 (75.4)	13 (72)	67 (76.1)	61 (67)	104 (63)	31 (58.5)	196 (63.4)	.02
Aromatase inhibitors + tamoxifen		0	13 (20)	4 (22)	17 (19.3)	25 (27.5)	48 (29)	18 (34)	91 (29.5)	
Aromatase inhibitors		0	1 (1.5)	0	1 (1.2)	5 (5.5)	10 (6)	3 (5.7)	18 (5.8)	
Unknown type		0	2 (3)	1 (5.5)	3 (3.4)	0	3 (2)	1 (1.8)	4 (1.3)	
With adjuvant chemotherapy‡ − no. of women (%)		3 (27.3)	51 (65.4)	20 (100)	74 (69)	3 (2.6)	28 (14.3)	42 (53.8)	73 (18.8)	<.0001
No anthracyclines		0	17 (33.3)	5 (25)	22 (35.6)	1	9 (32.1)	16 (38.1)	26 (29.7)	.88
Anthracyclines, no taxanes		3 (100)	29 (56.9)	12 (60)	44 (54.8)	1	16 (57.1)	23 (54.8)	40 (59.5)	
Anthracyclines and taxanes		0	2 (3.9)	2 (10)	4 (4.1)	0	0	3 (7.1)	3 (5.4)	
Unkown type		0	3 (5.9)	1 (5)	4 (5.5)	1	3 (10.8)	0	4 (5.4)	
Loco-regional recurrence event – no. of women (%)		0	4 (5.1)	2 (10)	7 (6.4)	1 (.86)	2 (1)	10 (12.8)	13 (3.3)	.14
Distant recurrence event – no. of women (%)		0	3 (3.8)	3 (15)	6 (5.5)	2 (1.7)	10 (5.1)	14 (17.9)	26 (6.7)	.65
Deaths – no. of women (%)		0	3 (3.8)	3 (15)	6 (5.5)	10 (8.6)	31 (15.8)	22 (28.2)	63 (16.2)	.004
Charlson co-morbidity score mean (SE, range)		0	0.10	0.05	0.08	0.11	0.20	0.19	0.18	.028
					(0.03, 0–2)				(0.03, 0–6)	
Charlson co-morbidity score – no. of women (%)										
0		11 (100)	71 (91)	19 (95)	101(92.6)	104 (90.4)	171 (87.3)	69 (88.4)	344 (88.4)	.86
1		0	6 (7.7)	1 (5)	7 (6.4)	9 (7.8)	18 (9.2)	6 (7.7)	33 (8.4)	
2		0	1 (1.3)	0	1 (1)	2 (1.8)	3 (1.5)	1 (1.3)	6 (1.5)	
3		0	0	0	0	0	2 (1)	1 (1.3)	3 (.8)	
4		0	0	0	0	0	0	1 (1.3)	1 (.3)	
5		0	0	0	0	0	1 (.5)	0	1 (.3)	
6	0	0	0	0		0	1 (.5)	0	1 (.3)	

* Categorization of a patient’s risk for recurrence as low, intermediate, or high was according to the Canadian clinical practice guidelines [4]. Low risk: Post-menopausal women with primary tumour size < 2 cm and tumour grade = 1; pre-menopausal women with primary tumour size < 1cm and tumour grade =1. High risk: All women with tumour size >3 cm, or women with tumour size ≥ 1 cm and ≤ 3 cm with tumour grade = 3. Intermediate risk: Post-menopausal women with tumour size < 2 cm and tumour grade > 1, or tumour size ≥ 2 cm and < 3 cm and tumour grade = 1 or 2; premenopausal women with tumour size < 1 cm and tumour grade >, or tumour size ≥ 1 cm and < 3 cm and tumour grade = 1 or 2. Given the significant correlation between tumour size, lymphatic and vascular invasion [35], and tumour grade [66], lymphatic and vascular invasion was not used in categorizing patients’ risk because the Manitoba cancer registry does not collect this information and 52 patients ‘risk for recurrence was categorized on the basis of tumour size only because their tumors size < 3 cm with undetermined tumours grade.

† The p-value was calculated for overall pre- vs. overall post-menopausal women. Fisher’s exact and chi-square tests were used for binary and categorical variables respectively. Distributions of continuous variables were summarized by their means and standard errors and compared using t-tests.

‡ Women were defined as having received any of these treatments for their primary breast cancer if the International Classification of Disease, Ninth Revision, Clinical Modification (ICD-9-CM) procedure code or the Canadian Classification of Health Interventions (CCI) procedure code of any of these treatments was found before any recurrence, second primary cancer or death within one year of diagnosis with ESBC.

Co-morbid diagnoses were considered present if they were found during one year before and 6 months after the diagnosis with primary breast cancer.

Anthracycline-containing regimens may have a survival advantage compared to CMF regimens [45]. However, other studies showed anthracycline-containing regimens to have equivalent clinical outcomes compared to CMF regimens, particularly in women with favourable prognostic features (LN-, ER+/PR+) such as our study cohort [4,46,47]. Thus, in sensitivity analysis we considered each of the CMF, AC, FAC and TAC regimens separately as the standard adjuvant chemotherapy regimen for women with ER+/ PR + LN- ESBC.

### Adjuvant chemotherapy-related serious adverse effects (CSAE)

We defined CSAE as hospitalization for any of the following eight diagnoses (as defined by their ICD-9-CM diagnosis and procedure codes) occurring within one year of diagnosis with ESBC: 1) abnormal electrolytes or dehydration; 2) constitutional symptoms and nonspecific symptoms associated with therapy; 3) nausea, emesis, and diarrhea; 4) infection and fever; 5) malnutrition; 6) anemia and red cell transfusion; 7) neutropenia or thrombocytopenia; 8) deep venous thrombosis or pulmonary embolus [48,49]. These diagnoses were selected based on their association with chemotherapy in previous clinical trials [3]. We estimated the incremental rate of occurrence of CSAEs from the frequency of occurrence of these ICD-9 codes in hospital abstracts of adjuvant chemotherapy recipients versus non-recipients, stratified by menopausal status and adjusting for comorbidity indices using the method developed by Charlson et al. excluding cancer diagnoses [50].

### Costs

Treatment costs, including surgery, radiation therapy, chemotherapy, endocrine therapy, surveillance, and CSAE, are all publicly funded in Manitoba and are thus recorded in the administrative databases. For each patient in the studied cohort we gathered all treatment costs for the first 7 years following diagnosis with primary breast cancer (Table 2). We used this to estimate the cost per unit time in each Markov state.

### Utilities

The baseline utility for post-menopausal women aged 50 to 80 was 0.824 and for premenopausal women aged 20 to 49 was 0.89, based on representative values for the U.S. population [51]. We derived utilities for each health state by multiplying these baseline utility values by utility estimates for women with early-stage breast cancer [41,52-55] (Table 2). We performed sensitivity analysis on the utility values after chemotherapy to account for potential long term side effects of primary adjuvant chemotherapy [56].

## Results

Patient, tumour, treatment and event characteristics of the study cohort are summarized in Table 3. There were 109 pre-menopausal and 389 post-menopausal women diagnosed with ER+/ PR + LN- ESBC in Manitoba from January 1, 2000 to December 31, 2002. The median age was 44 years (range 29–49 years) in pre-menopausal women and 62 years (range 50–88) in post-menopausal women. All pre- and post-menopausal women received surgery (mastectomy or breast-conserving surgery) for their primary breast cancer. Adjuvant therapy including radiation therapy, endocrine therapy (tamoxifen or aromatase inhibitors) and chemotherapy were administered in 63%, 81% and 69% of pre-menopausal women, respectively, and in 52%, 79% and 19% of post-menopausal women, respectively.

In pre-menopausal women, the RS-assay led to an increase of 0.05 QALY per person and decrease in cost of $50 per person resulting in a cost saving compared to CCP. In post-menopausal women, the RS-assay led to an increase of 0.062 QALY per person and an increase in cost of $3,700 per person, resulting in an incremental cost effectiveness ratio (ICER) of approximately $60,000 per QALY gained compared to CCP.

### Sensitivity analysis

In the base case we compared the RS assay versus CCP when the weighted average cost of CMF, AC and TAC was used. We considered each of CMF, AC, FAC and TAC regimens separately as the standard adjuvant chemotherapy regimen for women with ER+/ PR + LN- ESBC in sensitivity analysis. In premenopausal women, the RS-assay stayed cost saving with each of CMF, AC, FAC and TAC regimens. In post-menopausal women, the RS-assay had an ICER of $59,800 per QALY gained with CMF, $58,200 per QALY gained with AC, $65,000 per QALY gained with FAC and $83,100 per QALY gained with TAC. The utility during chemotherapy and the rates and costs of CSAE did not substantially influence the results with any regimen.

We performed threshold analyses on the proportion of chemotherapy-treated women classified as being in the intermediate risk group by the RS-assay, on the risk of relapse in the RS-assay model and other parameters found to influence our base case analyses (Tables 4 and 5). Among pre-menopausal women, the RS-assay generated negative incremental cost and effect (the RS-assay led to decrease in both cost and effect) and when fewer than 43% of women in the RS-assay intermediate risk group received adjuvant chemotherapy. Among postmenopausal women, the RS-assay was dominated by CCP when fewer than 31% of women in the RS-assay intermediate risk group received adjuvant chemotherapy. When the absolute risk of relapse in the RS-assay model increased by approximately 2% in either pre- or post-menopausal women, the RS-assay would be dominated by CCP or associated with negative incremental cost and effect.

**Table 4:** **Summary of important one-and two way sensitivity analyses**^**a**^

**Interpretation of the incremental impact of the RS-assay compared to CCP**							
**Variable (range tested)**		**Negative cost and effect**	**Cost saving**	**ICER in the range**	**ICER in the range**	**ICER in the range**	**Dominated**
				**0 to 20,000 $/QALY gained**	**20,000 to 100,000 $/QALY gained**	**>100,000 $/QALY gained**	
Chemotherapy treated women in intermediate risk group by the RS-assay (0% to 100%)		0% to 42%	43% to 63%	64% to 100%			
Change in absolute risk of relapse^b^ in the RS-assay model (−10% to +10%)		> +1.8%	≤ +1.8%				
Change in utility of recurrence^c^ (−10% to +10%)	Lower limit cost of recurrence^c^			≤ +2.2%	+2.3% to +3.4%	+3.5% to +4%	≥ +4%
	Baseline cost of recurrence^c^	> +3%	≤ +3%				
	Upper limit cost of recurrence^c^	> +3%	≤ +3%				
Change in utility following adjuvant chemotherapy (−10% to +10%)		> +1%	≤ +1%				

CMF = 6 cycles of cyclophosphamide, methotrexate, 5-fluorouracil; AC = 4 cycles of adriamycin, cyclophosphamide; CCP = current clinical practice.

^a^ Values in the table show how the incremental impact of the RS-assay compared to CCP changes, over 6 significant ranges, depending on the values of certain key parameters. For example, if between 43-63% of women identified as intermediate risk by the RS-assay were to receive chemotherapy, then the RS-assay would be cost saving relative to CCP; if this proportion is 64% or greater, then the RS-assay has an ICER between 0 and $20,000 / QALY gained.

^b^ Relapse includes loco-regional recurrence, distant recurrence and death due to any cause.

^c^ Recurrence includes loco-regional and distant recurrences.

**Table 5:** **Summary of important one-and two way sensitivity analyses**^**a**^

**Interpretation of the incremental impact of the RS-assay compared to CCP**							
**Variable (range tested)**		**Negative cost and effect**	**Cost savings**	**ICER in the range**	**ICER in the range**	**ICER in the range**	**Dominated**
				**0 to 20,000 $/QALY gained**	**20,000 to 100,000 $/QALY gained**	**> 100,000 $/QALY gained**	
Chemotherapy treated women in intermediate risk group by the RS-assay (0% to 100%)				86% to 100%	42% to 85%	32% to 41%	0% to 31%
Change in absolute risk of relapse^b^ in the RS-assay model (−10% to +10%)				< −3%	−3% to +0.9%	+1% to +2%	> +2%
Change in utility of recurrence^c^ (−10% to +10%)	Lower limit cost of recurrence^c^				< +9%	≥ +9%	
	Baseline cost of recurrence^c^				−10% to +10%		
	Upper limit cost of recurrence^c^				−10% to +10%		
Change in utility following adjuvant chemotherapy (−10% to +10%)				> 4.5%	−0.8% to +4.5%	−2.4% to −0.9%	≤ −2.5%

CMF = 6 cycles of cyclophosphamide, methotrexate, 5-fluorouracil; AC = 4 cycles of adriamycin, cyclophosphamide; CCP = current clinical practice.

^a^ Values in the table show how the incremental impact of the RS-assay compared to CCP changes, over 6 significant ranges, depending on the values of certain key parameters. For example, if between 42-85% of women identified as intermediate risk by the RS-assay were to receive chemotherapy, then the RS-assay has an ICER between $20,000 / QALY gained and $100,000 / QALY gained; if this proportion is between 32% and 41%, then the RS-assay has an ICER greater than $100,000 / QALY gained.

^b^ Relapse includes loco-regional recurrence, distant recurrence and death due to any cause.

^c^ Recurrence includes loco-regional and distant recurrences.

**Figure 2:**
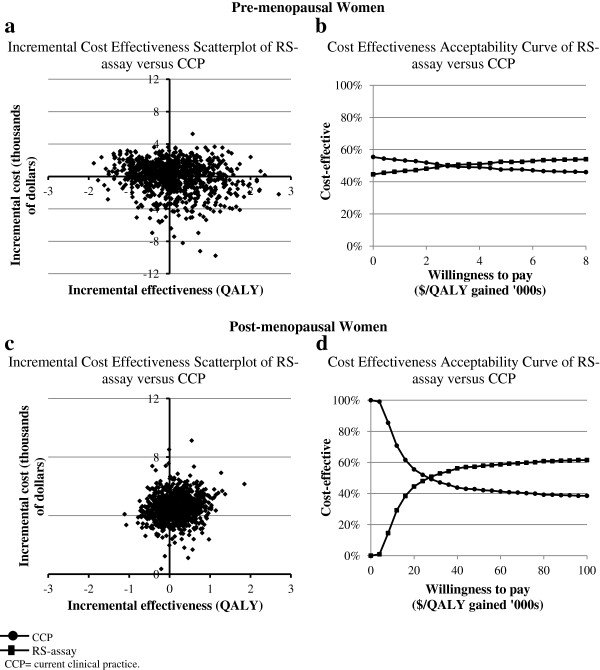
**Incremental cost-effectiveness scatterplot and acceptability curve of RS-assay-guided therapy versus CCP-guided therapy for pre- and post-menopausal women.** Sampling distributions and summary estimates of cost, efficacy, and variance were based on 1000 replicates.

We also performed a probabilistic sensitivity analysis (Figure 2) comparing the RS-assay versus CCP. We simultaneously varied all parameters (probabilities, utilities and costs) using appropriate distributions (Table 2). In pre-menopausal women, using a willingneess to pay threshold of $100,000 per QALY gained, we found that the RS-assay was the prefered strategy in 54% of simulations (Figure 2a and b). In post-menopausal women, we found that the RS-assay was the prefered strategy in 62% of simulations (Figure 2c and d).

## Discussion

We developed a decision-analytic model to evaluate the cost effectiveness of the RS-assay versus CCP in ER+/ PR + LN- ESBC. In the base case we estimated that the RS-assay generated cost savings in pre-menopausal women and has an ICER of $60,000 per QALY gained in post-menopausal women.

In Canada, an ICER threshold of $100,000 per QALY gained has been suggested as representing “weak evidence for adoption and appropriate utilization” [27,57], although there is no evidence that any Canadian decision-making body has formally implemented this threshold [58]. The ICERs of the RS-assay in post-menopausal women were within ranges of a number of cancer treatments that have recently been approved in Canada. For instance, sorafinib has an estimated ICER of $75,821 per life year gained for the treatment of hepatocellular carcinoma and has been approved for funding in Ontario through the Exceptional Access Program [59]. Sunitinib has been funded in all Canadian provinces for first-line treatment of metastatic renal-cell carcinoma with an ICER of $144,000 per QALY gained [60].

Previous cost-effectiveness analyses of the RS-assay in ER+/ PR + LN- ESBC population have several limitations and may not be applicable in the Canadian context. One study [18] did not incorporate results from NSABP B20 [9], which established the relationship between the RS-assay and the benefit from using chemotherapy. Another two studies [19,20] included results from NSABP B20 [19]; however, the treatment strategies that they compared (tamoxifen alone for everyone and tamoxifen and chemotherapy for everyone) do not reflect observed clinical practice in Canada (Table 3). Other studies from Israel [22] and Japan [21] did not incorporate all early stage breast cancer complications such as local or regional recurrence. Two recent studies [23,24] were conducted from the Canadian health care payer’s perspective; however, the first analysis [23] did not address all the limitations mentioned above, and modeling the current experience of ER+/ PR + LN- ESBC population with regard to survival in both analyses [23,24] was not based on Canadian data and real world clinical practice. In all studies there was no differentiation in adjuvant chemotherapy practice between pre- and post-menopausal women as recommended by Canadian guidelines [4], whereas we observed differences in clinical practice for these two groups (Table 3).

Adjuvant chemotherapy is a widely recommended treatment in ER+/ PR + LN- ESBC [15]. Thus, some have suggested that large cost savings can be expected by avoiding chemotherapy treatment in 25% to 35% of patients based on the results of the RS-assay [15]. Our analysis suggests that cost savings may be possible in pre-menopausal women, due the wide use of chemotherapy in this group, but would likely not occur with post-menopausal women with ER+/ PR + LN- ESBC.

In sensitivity analysis we addressed the economic impact of uncertainty in clinical guidelines for intermediate-range RS-assay values (18–30) [61]. Our analysis demonstrated that the ability of the RS-assay to guide treatment decisions in the intermediate risk group likely will be important in determining whether the RS-assay will be a cost-effective use of resources. If fewer than 43% of pre-menopausal and 31% of post-menopausal women identified as intermediate risk by the RS-assay received adjuvant chemotherapy, then the RS-assay had negative health effects compared to CCP. An ongoing prospective clinical trial will further assess the predictive value of the assay in women in the intermediate risk group and will be helpful in verifying our results [10]. However, findings from this trial will not be available for 5 to 10 years whereas an adoption decision will need to be made prior to having the results of this trial.

Our analysis has several limitations. First, there are limits to what can be ascertained through administrative data. Although the Manitoba Cancer Registry is a highly accurate source of information about breast cancer [28], errors in coding can result in incorrect or unrecorded procedures. However, wherever possible we cross validated across databases. For instance, information on breast cancer treatments including surgery, radiation therapy, endocrine therapy and chemotherapy can be found in both the Manitoba Cancer Registry and the administrative databases held by Manitoba Health and Healthy Living. Second, validation data for the 21-gene assay was based on retrospective analyses of the NSABP chemotherapy-tamoxifen trials (B-14 and B-20) conducted in the United States [9,11]. Thus, survival outcomes by the RS-assay may not reflect the experience of the ER+/PR + LN- ESBC identified in Manitoba due to possible differences in patient and tumour characteristics and treatments. Results from future prospective analyses of the assay in real-world clinical practice and in Canadian settings can be used to update our model and verify our results. Third, there is still uncertainty as to whether chemotherapy is necessary for women who fall in the intermediate risk group by the RS-assay [10]. Fourth, newer third generation anthracycline-taxane regimens have different costs and slightly better efficacy so analysis with such data would be more applicable to the current practice landscape. In addition, our analysis did not account for growing data on long term side effects of primary adjuvant chemotherapy such as cardiomyopathy, neuropathy, leukemia [56]. Finally, although several studies have found that clinical practice patterns and therapies employed in the selected time periods in Manitoba reflect practice in other jurisdictions in Canada [62-64], differences in clinical practice for women with ER+/PR + LN- ESBC and its associated costs across Canadian provinces may still exist.

## Conclusions

We compared the RS-assay versus current clinical practice in ER+/ PR + LN- ESBC for both pre- and post-menopausal women. We found that it is likely to be cost-saving for pre-menopausal women and to have an ICER that is within ranges of a number of cancer treatments recently approved for funding in Canada for post-menopausal women. Validation of the assay in real-world clinical practice is warranted to verify the retrospective analyses of this assay in clinical trials and ensure its cost-effectiveness for routine use in this population.

## Competing interest

The authors declare that they have no competing interest.

## Authors' contributions

MBH designed the study, performed the statistical analysis and drafted the manuscript. BX participated in the design of the study and coordination. MB participated in the design of the study and coordination. GSZ participated in the design of the study, participated in statistical analysis and drafting the manuscript. All authors read and approved the final manuscript.

## Funding

This work was supported by the Canadian Institutes of Health Research (CIHR) Strategic Training Program in Cancer Research and Technology Transfer (CaRTT); an Academic Development Grant from the University of Western Ontario; and the Natural Sciences and Engineering Research Council of Canada (NSERC) GSZ was supported by the Canada Research Chairs Program. The authors’ work was independent of the funders and the funding sources had no involvement in the study design or conduct of the study; collection, management, analysis or interpretation of the data; or preparation, review or approval of the manuscript.

## Pre-publication history

The pre-publication history for this paper can be accessed here:

http://www.biomedcentral.com/1471-2407/12/447/prepub
